# Association between Vitamin D Status and Risk of Metabolic Syndrome among Korean Postmenopausal Women

**DOI:** 10.1371/journal.pone.0089721

**Published:** 2014-02-21

**Authors:** Seung Joo Chon, Bo Hyon Yun, Yeon Soo Jung, Si Hyun Cho, Young Sik Choi, Suk Young Kim, Byung Seok Lee, Seok Kyo Seo

**Affiliations:** 1 Department of Obstetrics and Gynecology, Severance Hospital, Yonsei University College of Medicine, Seoul, South Korea; 2 Department of Obstetrics and Gynecology, Gangnam Severance Hospital, Yonsei University College of Medicine, Seoul, South Korea; 3 Department of Obstetrics and Gynecology, Gil Hospital, Gachon University, Incheon, South Korea; Innsbruck Medical University, Austria

## Abstract

This study aimed to investigate the association between serum levels of 25-hydroxyvitamin D [25(OH)D] and metabolic syndrome along with its associated risk factors in Korean postmenopausal women. This study was performed using data from the KNHANES 2008–2010 study and included 4,364 postmenopausal Korean women. Clinical and other objective characteristics, seasonality, and presence of metabolic syndrome with its five components were evaluated and correlated with the serum levels of 25(OH)D. Although no statistically significant associations were observed between the levels of serum 25(OH)D and the prevalence of metabolic syndrome, the adjusted OR for elevated blood pressure, elevated triglycerides (TGs), and reduced high-density lipoprotein cholesterol (HDL-C) showed tendency to decrease sequentially as tertiles of serum 25(OH)D levels increased (*p* for trends  = 0.066, 0.043, and 0.010, respectively). Women in the highest tertile of serum 25(OH)D showed a significant decrease in the prevalence of elevated blood pressure, elevated TGs, and reduced HDL-C as compared with those in the lowest tertile of serum 25(OH)D (*p* = 0.020, 0.014, and 0.002, respectively). Based on these results, we consider that adequate serum levels of 25(OH)D in Korean postmenopausal women may not entirely indicate a lower risk of developing metabolic syndrome. However, adequate serum levels of 25(OH)D are significantly associated with a decrease in elevated blood pressure, elevated TGs, and reduced HDL-C levels in postmenopausal women.

## Introduction

Cardiovascular complications are the major causes of death in postmenopausal women. This is important because approximately 95% of women in urban areas experience menopause for more than one-third of their lives [1]. Since menopause is a risk factor for cardiovascular disease, it should be viewed as one of the most serious problems faced by women. When women experience menopause, they should not only be aware of preexisting cardiovascular risk factors, but they should also closely observe their overall health to help prevent cardiovascular disease.

Metabolic syndrome is a clinical disorder characterized by the co-occurrence of heterogeneous traits, including abdominal obesity, hypertension, dyslipidemia (high triglycerides (TG) and low high-density lipoprotein cholesterol (HDL-C) levels), and impaired glucose tolerance [2]. It is a well-recognized cluster of modifiable risk factors of cardiovascular disease and type 2 diabetes mellitus (DM) [3], [4]. The number of individuals with metabolic syndrome is increasing in Asia [5], especially due to the increased consumption of a westernized diet and reduced physical activity [4].

Evidence suggests that maintaining vitamin D levels might provide protective effects against metabolic syndrome and its sequelae [6]. The prevalence of metabolic syndrome is known to increase during menopause; however, studies evaluating the prevalence of metabolic syndrome and its association with serum levels of vitamin D in postmenopausal women are lacking. Therefore, managing metabolic syndrome and studying its risk factors, particularly in postmenopausal women, is crucial for improving health in this population. Since the serum levels of vitamin D and the prevalence of metabolic syndrome vary by ethnicity [7], it is important to study this association in postmenopausal women in the Korean population.

The aim of this study was to evaluate the prevalence of vitamin D deficiency and metabolic syndrome in postmenopausal Korean women. Additionally, by grouping participants into tertiles based on serum 25-hydroxyvitamin D [25-(OH)D] levels, we were able to assess the effects of menopausal status on metabolic syndrome and its associated factors among Korean postmenopausal women.

## Methods

### Study Population

This study was performed using data from the Korean National Health and Nutrition Examination Survey (KNHANES) (2008–2010 data), specifically data from the KNHANES IV survey (data from 2008 and 2009) and the KNHANES V survey (data from 2010), all performed by the Korean Ministry of Health and Welfare. KNHANES IV and V were each conducted for 3 years (2007–2009 and 2010–2012, respectively), using a rolling sampling survey that involved a complex, stratified, multistage, probability-cluster survey of a representative sample of the non-institutionalized civilian population in South Korea. Sampling units were randomly selected, with 23 households from each primary sampling unit, with 200 randomly selected sampling units, yielding 4600 households in 2008, whereas 192 sampling units were randomly selected, with 20 households from each primary sampling unit, yielding 3840 households in 2009 and 2010. The survey was composed of three parts: a health interview survey, a health examination survey, and a nutrition survey. Each survey was conducted by specially trained interviewers. The interviewers were not provided with any prior information regarding specific participants before conducting the interviews. Participants provided written informed consent to participate in this survey, and we received the data in anonymized form. The study was approved by the Yonsei University Health System, Severance Hospital, Institutional Review Board (4–2013–0393).

We excluded male participants and individuals who were pregnant or had chronic liver or renal disease. In addition, participants were excluded if they had not provided blood samples for 25(OH)D, fasting plasma glucose (FPG), and lipid profiles including TG and HDL-C; or had not completed the self-reporting questions regarding district resident type, smoking, alcohol consumption, physical activity, education level, occupation, income, diet, sampled seasonality, use of anti-hypertensive, diabetic, or anti-dyslipidemic medications, and use of hormone therapy (HT). We also excluded subjects who did not provide anthropometric measurements, including blood pressure (BP; systolic and diastolic, i.e., systolic BP [SBP] and diastolic BP [DBP], respectively), height, weight, and waist circumference (WC) as well as women who had undergone surgical menopause. Eventually, 10,907 (6,543 premenopausal and 4,364 postmenopausal women) women were classified based on the presence of menopause, and 4,364 postmenopausal women were enrolled in the present study.

### Variable Measurements

Based on age, menopausal women were grouped into 6 categories. District residents were categorized as urban and rural. Seoul (the capital city of Korea), its surrounding capital area (Gyonggi), and six other metropolitan cities (Incheon, Daejeon, Gwangju, Daegu, Busan, and Ulsan) were grouped as urban areas, and the remaining regions of South Korea were grouped as rural areas. People were classified as current smokers, if they had smoked at least one cigarette per day during the previous 12 months. Alcohol consumption was evaluated and categorized into two groups, depending on the history of drinking for the previous 12 months. Based on the International Physical Activity Questionnaire short-form scoring protocol, physical activity levels were divided into three categories as low, moderate, and high [8]. Education level was classified into three categories based on the educational background: (1) elementary school; (2) middle or high school; and (3) college or university. Occupation was classified into four categories; (1) people who work regularly in an office, (2) people who usually work outdoors, (3) people who work on a farm or in a fishing village, and (4) people who usually stay at home. Household income was divided into quartiles according to the mean household income per month (income per month/√number of family members). The circumstances of oral intake were classified into three groups to predict whether they had been supplied with sufficient nutrition (both quantity and quality) through their meals as follows: (1) insufficient quantity and quality frequently, (2) insufficient quantity and quality occasionally, and (3) sufficient quantity with diverse nutritional supplementations. The seasons in which the blood samples were taken were classified into four categories as follows: (1) spring: March to May, (2) summer: June to August, (3) fall: September to November, and (4) winter: December to February. The usage of HT was divided into two categories as “ever” and “never” users.

Anthropometric measurements were performed. Height and weight were measured with subjects wearing light clothing but without shoes. Waist circumference was measured at the midpoint between the lower costal margin and iliac crest at the end of normal expiration. Blood pressure was measured in the sitting position after resting for 10 min, twice with 5-min intervals, and the average value in mmHg was used. Blood samples were collected early in the morning after an overnight fast. Plasma concentrations of glucose, TGs, and HDL-C were measured following routine biochemical laboratory protocols. Serum 25(OH)D levels were determined by electrochemiluminescence immunoassay using a Cobas autoanalyzer (Roche Diagnostics, West Sussex, UK; intra- and interassay coefficient of variations (CVs) <8% and <10%, respectively). All clinical analyses were performed by the Neodin Medical Institute, a laboratory certified by the Korean Ministry of Health and Welfare.

### Criteria and Definitions

Menopause is defined as amenorrhea for 12 months following the final menstrual period [9]. In our study, postmenopausal status was defined as the self-reported cessation of menstruation for more than 1 year, and we excluded women who had undergone a hysterectomy or bilateral salpingo-oophorectomy (BSO).

Although there is no consensus regarding the optimal serum levels of 25(OH)D, in order to compare the vitamin D status in various age groups among postmenopausal women, we classified it as deficiency, insufficiency, and sufficiency as follows. Based on previous data, vitamin D deficiency was defined as serum 25(OH)D levels less than 20 ng/mL [10]; vitamin D insufficiency, as ≥20 ng/mL to <30 ng/mL; and vitamin D sufficiency, as ≥30 ng/mL [11]. We also grouped the participants according to the tertiles of 25(OH)D (group 1 as 3.070–14.890 ng/mL, group 2 as 14.900–20.969 ng/mL, and group 3 as 20.970–66.960 ng/mL) in order to compare baseline characteristics and to examine the relationship between vitamin D status and the prevalence of metabolic syndrome along with the associated factors among the groups.

Metabolic syndrome was defined as the presence of three or more of the following: (1) abdominal obesity (waist circumference ≥85 cm in women according to the Korean Society for the study of Obesity), (2) elevated blood pressure (average systolic blood pressure ≥130 mmHg or diastolic blood pressure ≥85 mmHg) or currently undergoing treatment for hypertension, (3) elevated serum triglycerides (≥150 mg/dL) or current drug treatment for high TGs, (4) reduced HDL cholesterol (<50 mg/dL) or current drug treatment for low HDL-C, and (5) elevated fasting glucose levels (≥100 mg/dL) or current usage of hypoglycemic agents or insulin [12].

### Statistical Analysis

Data are presented as means [95% confidence interval (CI)] for continuous variables and percentages for categorical variables unless otherwise stated. The status of vitamin D, the prevalence of metabolic syndrome according to age groups, and baseline characteristics depending on serum vitamin D levels were calculated using chi-square test (χ^2^ test). For the analysis of differences between the prevalence of metabolic syndrome and its risk factors among the three groups with different vitamin D levels, one-way analysis of variance (ANOVA) was used for continuous data, and χ^2^ test was used for categorical data.

After confirming that the values of serum levels of 25(OH)D were normally distributed, the odds ratio (OR), *p* for trends for the prevalence of metabolic syndrome along with its factors according to the tertiles of serum 25(OH)D, and *p*-value for the prevalence of metabolic syndrome with its associated factors between the lowest and the highest tertiles of serum 25(OH)D were analyzed using linear logistic regression. The covariates for the adjusted OR calculation included age, seasonality, occupation, education, alcohol, smoking, physical activity, and HT.

Data analysis was carried out using SPSS software (version 20; SPSS, Chicago, IL), and *p*<0.05 was considered statistically significant.

## Results

**Figure 1:**
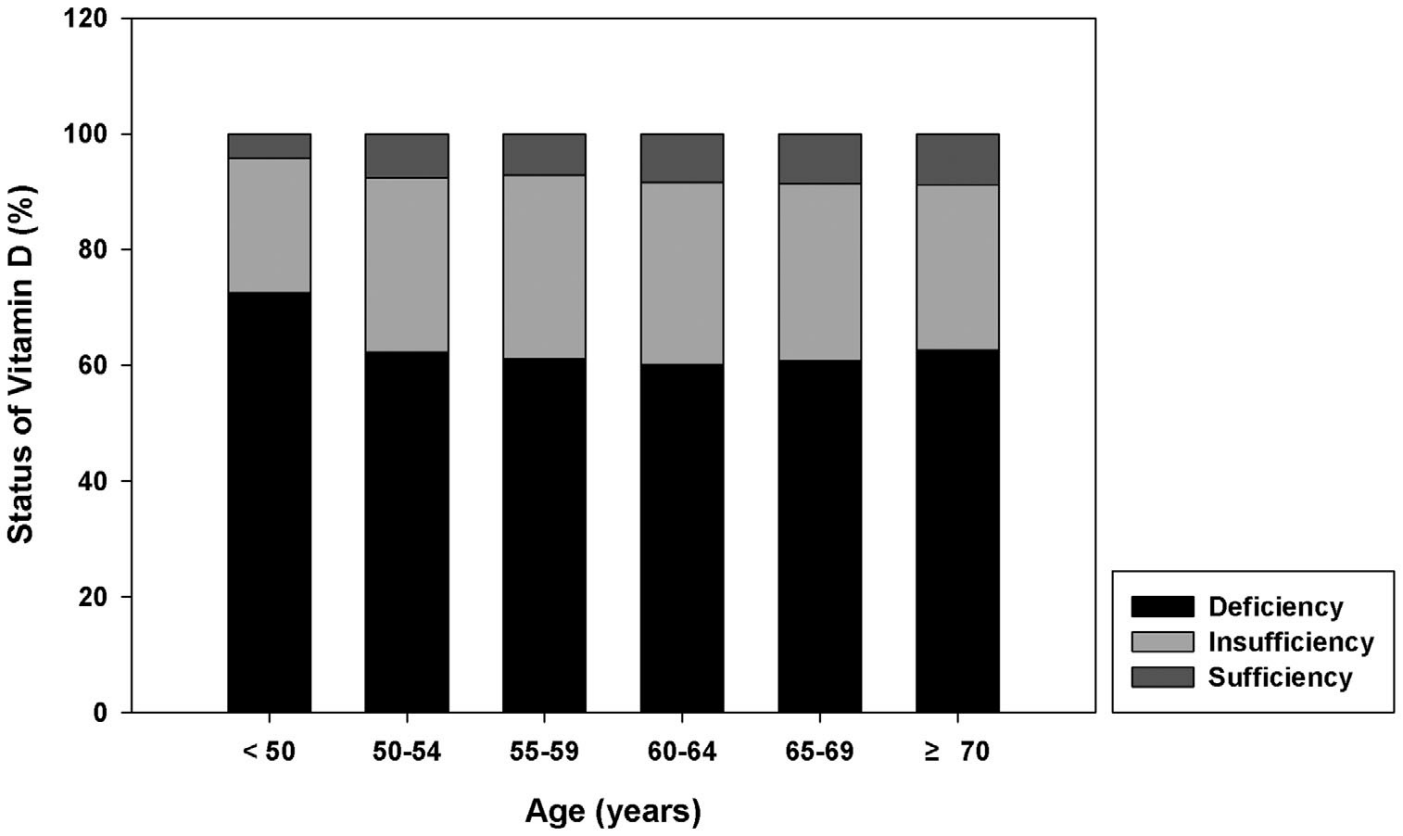
Vitamin D status according to age groups in postmenopausal women. The lowest portion of the graph represents vitamin D deficiency; the middle portion, vitamin D insufficiency; and the highest portion, vitamin D sufficiency. Vitamin D deficiency was dominant in the postmenopausal South Korean female population (62.1%). The prevalence of vitamin D deficiency was the highest in group of <50 years, 72.5% and the lowest in group of 60–64 years, 60.1%.

**Figure 2:**
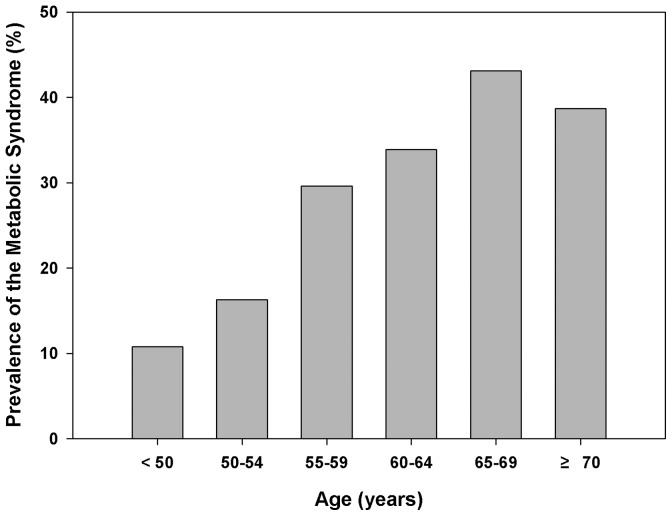
Prevalence of metabolic syndrome in postmenopausal women. The prevalence of metabolic syndrome increased from 10.8% in women younger than 50 years to 43.1% in those aged 65–69 years.

Figures 1 and 2 show the vitamin D status and the prevalence of metabolic syndrome in postmenopausal Korean women. Vitamin D deficiency was dominant in the general South Korean postmenopausal female population (62.1%). The prevalence of vitamin D deficiency was the highest in a group of <50 years and the lowest in a group of 60–64 years, being 72.5% and 60.1%, respectively (Figure 1). The prevalence of metabolic syndrome increased from 10.8% in women from a group of <50 years to 43.1% in a group of 65–69 years (Figure 2).

Table 1 shows the baseline characteristics of 4364 postmenopausal women according to tertile groups of serum 25(OH). For increasing serum 25(OH)D levels, the percentages of people living in urban areas tended to decrease whereas people in rural area increased (*p*<0.001); people with low physical activity showed lower percentages of proportion in the higher tertiles, whereas those with high physical activity tended to show higher percentages of proportion (*p*<0.001). Moreover, subjects who worked outdoors (farming or fishing) showed the highest proportions in the highest tertiles of serum 25(OH)D, whereas those mostly working indoors (office workers and housewives) showed the highest proportions in the lowest tertiles of serum 25(OH)D (*p*<0.001). Most importantly, people sampled in the spring or winter were present in the highest proportions in the lowest tertiles of serum 25(OH)D, while those sampled in the summer or autumn had the highest proportions in the highest tertiles of 25(OH)D (*p*<0.001).

**Table 1:** General characteristics of postmenopausal participants compared by vitamin D status (n = 4364).

			Serum 25(OH)D		
		Group 1 (%)	Group 2 (%)	Group 3 (%)	*p*-value
Number		1454	1456	1454	
Residents district	Urban	992 (68.2)	811 (55.7)	742 (51.0)	<0.001
	Rural	462 (31.8)	645 (44.3)	712 (49.0)	
Smoking	Never	1307 (90.0)	1314 (90.4)	1338 (92.0)	0.351
	Ever	24 (1.7)	26 (1.8)	19 (1.3)	
	Current	122 (8.4)	113 (7.8)	97 (6.7)	
Alcohol consumption	None	1238 (85.2)	1202 (82.7)	1219 (83.8)	0.190
	Yes	215 (14.8)	251 (17.3)	235 (16.2)	
Physical activity	Low	1169 (80.6)	1125 (77.6)	1064 (73.3)	<0.001
	Moderate	128 (8.8)	151 (10.4)	180 (12.4)	
	High	154 (10.6)	173 (11.9)	208 (14.3)	
Education level	Elementary	937 (64.6)	943 (65.1)	984 (67.9)	0.067
	Middle/High	415 (28.6)	416 (28.7)	400 (27.6)	
	College/University	98 (6.8)	90 (6.2)	65 (4.5)	
Occupation	Office workers	88 (6.1)	72 (5.0)	42 (2.9)	<0.001
	Salesmen	134 (9.3)	152 (10.5)	107 (7.4)	
	Farmers/Fishermen	278 (19.2)	389 (26.8)	513 (35.4)	
	Housewives	947 (65.4)	836 (57.7)	786 (54.3)	
Income	Low	325 (23.1)	352 (24.5)	378 (26.5)	0.521
(quartile/person)	Medium-Low	365 (26.0)	363 (25.3)	358 (25.1)	
	Medium-High	356 (25.3)	375 (26.1)	349 (24.4)	
	High	360 (25.6)	347 (24.1)	343 (24.0)	
Diet	Frequent deficiency	79 (5.4)	68 (4.7)	68 (4.7)	0.857
	Occasional deficiency	896 (61.6)	896 (61.5)	896 (61.6)	
	Enough	479 (32.9)	492 (33.8)	490 (33.7)	
Seasonality	Spring	532 (36.6)	356 (24.5)	181 (12.4)	<0.001
	Summer	267 (18.4)	377 (25.9)	513 (35.3)	
	Autumn	242 (16.6)	378 (26.0)	544 (37.4)	
	Winter	413 (28.4)	345(23.7)	216 (14.9)	

The tertile groups according to the serum 25(OH)D levels were compared in terms of metabolic syndrome and its risk factors among postmenopausal women in Table 2. The values of SBP, DBP, and TGs in the lowest tertile of serum 25(OH)D were higher, and the value of HDL-C in the lowest tertile of serum 25(OH)D was lower than those in the highest tertile of 25(OH)D, and the differences were statistically significant (*p* = 0.044, 0.020, 0.001, and 0.005, respectively). Elevated TGs and reduced HDL-C also showed the highest statistically significant proportions in the lowest tertile of 25(OH)D (*p* = 0.031 and 0.004, respectively).

**Table 2:** Metabolic syndrome and its risk factors of postmenopausal participants compared by vitamin D status (n = 4364).

		Serum 25(OH)D		
	Group 1	Group 2	Group 3	*p*-value
WC (cm)	82.6±9.6	82.4±9.0	82.1±9.3	0.375
Abdominal obesity	564 (38.8)	553 (38.0)	552 (38.0)	0.872
SBP (mmHg)	127.5±18.8	127.2±18.2	125.9±18.0	0.044
DBP (mmHg)	77.4±10.1	77.9±10.2	76.9±9.9	0.020
Elevated BP or medication	905 (62.2)	871 (59.8)	850 (58.5)	0.108
FPG (mg/dL)	101.5±25.7	100.9±24.9	100.5±22.6	0.520
Elevated FPG or medication	547(37.6)	501 (34.4)	515 (35.4)	0.182
TGs (mg/dL)	143.2±88.4	137.1±88.2	131.6±80.2	0.001
Elevated TGs or medication	596 (41.0)	542 (37.2)	532 (36.6)	0.031
HDL-C (mg/dL)	51.3±12.4	52.4±12.3	52.7±12.7	0.005
Reduced HDL-C or medication	801 (55.1)	725 (49.8)	722 (49.7)	0.004
Metabolic syndrome	498 (34.3)	453 (31.1)	460 (31.6)	0.153

Data are presented as mean ± standard deviation; numbers (%).

Abbreviations: WC, waist circumference; SBP, systolic blood pressure; DBP, diastolic blood pressure; BP, blood pressure; FPG, fasting plasma glucose; TGs, triglycerides; HDL-C, high-density lipoprotein cholesterol.

Age, season, occupation, education, alcohol, smoking, physical activity, and HT-adjusted OR for metabolic syndrome and its components were evaluated according to the serum levels of 25(OH)D, which was categorized into tertile groups (Table 3). Among the three groups, as serum levels of 25(OH)D increased, no statistically significant associations were shown with the prevalence of metabolic syndrome (*p* for trends  = 0.333).

**Table 3:** Odds ratio of metabolic syndrome and its components by serum 25(OH)D levels in postmenopausal women (n = 4364).

		Serum 25(OH)D			
	Group 1	Group 2	Group 3	*p* for trends§	*p*-value†
	1445	1445	1446		
Metabolic syndrome	1.0	0.91 (0.77–1.06)	0.90 (0.76–1.05)	0.333	0.165
Abdominal obesity	1.0	0.98 (0.84–1.14)	0.95 (0.82–1.11)	0.825	0.538
Elevated BP	1.0	0.95 (0.81–1.12)	0.83 (0.71–0.98)	0.066	0.020
Elevated fasting glucose	1.0	0.89 (0.76–1.04)	0.90 (0.77–1.06)	0.288	0.259
Elevated TGs	1.0	0.87 (0.75–1.01)	0.83 (0.71–0.97)	0.043	0.014
Reduced HDL-C	1.0	0.84 (0.72–0.97)	0.80 (0.69–0.93)	0.010	0.002

Data are presented as OR (95% CI).

§ Values from comparisons of metabolic syndrome and its components among tertiles of 25(OH)D.

† Values from comparisons of metabolic syndrome and its components in between the lowest and highest tertiles of 25(OH)D.

Abbreviations: OR, odds ratio; CI, confidence interval; TGs, triglycerides; HDL-C, high-density lipoprotein cholesterol.

Adjusted for age, seasonality, occupation, education, alcohol, smoking, physical activity, and HT.

However, when we examined the components of metabolic syndrome, the OR for the prevalence of elevated BP, elevated TGs, and reduced HDL-C showed a tendency to decrease as the serum levels of 25(OH)D increased (*p* for trends  = 0.066, 0.043, and 0.010, respectively). Additionally, when the lowest and highest tertiles of serum 25(OH)D were compared, statistical significance was observed for the same components, i.e., elevated BP, elevated TGs, and reduced HDL-C (*p* = 0.020, 0.014, and 0.002, respectively).

## Discussion

In this study, we investigated whether serum levels of 25(OH)D have associations with the prevalence of metabolic syndrome along with its risk factors in postmenopausal Korean women. In this retrospective study, although the prevalence of metabolic syndrome in postmenopausal women tended to decrease as serum levels of 25(OH)D increased, this association was not statistically significant. However, we confirmed that higher serum levels of 25(OH)D were associated with a statistically significant decrease in the prevalence of the components of metabolic syndrome, i.e., elevated BP, elevated TGs, and reduced HDL-C.

Several reports have demonstrated a significant inverse association between serum 25(OH)D levels and the prevalence of metabolic syndrome [13]–[16]. However, other studies, similar to ours, have observed a lack of association between serum levels of 25(OH)D and metabolic syndrome. In a Korean study, no association was found between vitamin D deficiency and the overall metabolic syndrome risk [17]. In another study from South Africa, serum levels of 25(OH)D were not an independent predictor of metabolic syndrome in Africans and Asian Indians [18].

Several studies have examined the effects of menopause on metabolic syndrome, showing varied age groups for peak prevalence in diverse ethnicities. A higher prevalence of metabolic syndrome has been reported in women, especially those aged more than 50 years, as compared with men in a Korean study [19]. Another Korean study reported that the prevalence of metabolic syndrome increased after menopause [20]. A study in the U.S. demonstrated an increased risk of metabolic syndrome up to more than 20% among postmenopausal women [21], while another study from the U.S. demonstrated that postmenopausal status was consistently associated with an increased risk for metabolic syndrome, based on the National Health and Nutrition Examination Survey III [22]. Moreover, a study that examined the prevalence of metabolic syndrome and its association with hyperinsulinemia in the urban Korean population concluded that the prevalence of metabolic syndrome increased with increasing tertiles of insulin resistance [23]. However, to the best of our knowledge, our study is the first to demonstrate the effect of serum levels of 25(OH)D on metabolic syndrome among postmenopausal Korean women based on KNHANES.

The major factors leading to the results observed in the present study are the significant abnormalities in lipid profiles [5]. In our study, the adjusted OR for elevated TGs and reduced HDL-C decreased as the tertiles of serum 25(OH)D increased. In several studies, TGs [24] are generally known to be elevated in postmenopausal women as compared with premenopausal women, and the cardioprotective features in women are known to be lost after menopause with a significant decrease in HDL-C [25]. Although the mechanisms underlying the relationship between vitamin D status and dyslipidemia are not well known, one animal study reported that the plasma vitamin D concentration was positively associated with HDL-C (*p* = 0.003), concluding that lower vitamin D would be associated with a more atherogenic lipid profile, which is a major risk factor for progression toward coronary artery atherosclerosis [26]. In a process called reverse cholesterol transport, large HDL particles are known to carry cholesterol from atherosclerotic plaques [27], and these large HDL particles are driven from cholesterol-loaded macrophages by cholesterol efflux that it is vitamin D which regulates macrophage function [28].

Our result showed that elevated blood pressure was statistically significantly related with vitamin D status in postmenopausal Korean women. Similar results have been found in many clinical studies [29], [30]. Vitamin D receptors are distributed on vascular smooth muscle, endothelium, cardiomyocytes, and activated 1,25-dihydroxyvitamin D suppresses renin gene expression, regulating the growth and proliferation of vascular smooth muscle cells, cardiomyocytes, and inhibiting cytokine release from lymphocytes. Therefore, the absence of vitamin D receptor activation leads to tonic upregulation of the renin-angiotensin system, eventually leading to hypertension and left ventricular hypertrophy [31]. However, despite strong physiological evidence, some studies evaluating the association between serum levels of 25(OH)D and blood pressure have presented contradictory findings [32], [33].

Natural menopause is known to be associated with increased central adiposity [24]; further, in cases of similar mean BMI values for premenopausal and postmenopausal women, increased WC has been found to be significantly associated with postmenopausal status after adjusting for age [20]. A previous study reported that serum 25(OH)D levels were negatively associated with WC [34]. In our study, WC, the prevalence of abdominal obesity, and the OR for prevalence of abdominal obesity related to vitamin D increased as tertiles of 25(OH)D decreased; however, these results were not statistically significant. This could possibly be explained by a cultural tendency in Korean women, who are sensitive about their appearance and make the effort to maintain their physical appearance. Further, elevated FPG was not found to be related with serum levels of 25(OH)D, possibly because the overall plasma glucose level in this population was low.

As the numbers of UV photons reaching the earth's surface vary markedly by the time of the day and the season, vitamin D production in the skin decreases in the early morning, late afternoon, and during winter [17]. Although South Korea is located at the latitudes of 33–38,° which receive adequate numbers of UVB photons for synthesizing vitamin D [17], we have found a high prevalence of vitamin D deficiency in South Korean postmenopausal women, which could be explained by increased indoor activity and the use of sunscreens and other sun protectors. Since cutaneous vitamin D production in elderly individuals is known to diminish [35], it is easy to assume that the prevalence of vitamin D deficiency in women with older age would be higher. However, our results demonstrated that the prevalence of vitamin D deficiency was not directly proportional with age. This finding could be explained by an age-related decline in kidney function. A previous study in rats reported that as kidney function declines, so does 1,25(OH)_2_D production [28] as well as the metabolic clearance of 25(OH)D. Therefore, although vitamin D production in the skin decreases with age, so does the utilization of 25(OH)D in the kidney; therefore, these effects negate each other such that the serum levels of 25(OH)D are not greatly affected.

This study has several strengths. First, the study was performed using a representative sample of the general South Korean population. Second, rigorous quality controls were applied to the study procedures in KNHANES. Third, the exclusion of women who had undergone a hysterectomy reduced some bias because a certain proportion of such subjects might not have an accurate menopausal status. However, our study has certain limitations. First, because it is a cross-sectional study, direct associations between the variables of interest could not be determined. Second, we could not consider factors such as amounts of sunlight exposure, calcium intake, and vitamin D intake – which could have affected the serum levels of 25(OH)D – because of the limited data. Further, measurements were performed only once for each participant, and serial measurements over a year would be required for more accurate studies.

In summary, vitamin D deficiency was found to be common in South Korean menopausal women, showing a strong seasonal effect. Further, serum 25(OH)D levels were not significantly correlated with the prevalence of metabolic syndrome in postmenopausal women; however, elevated BP, elevated TG, and reduced HDL-C levels were found to decrease significantly as the serum levels of 25(OH)D increased. These findings demonstrate that metabolic syndrome is not entirely dependent upon its five components and is likely affected by other factors such as abnormalities in molecular pathophysiology, which are correlated with the prevalence of metabolic syndrome. Further investigations regarding these aspects would clarify such underlying factors.

## References

[1] Sitruk-Ware R, Ibarra de Palacios P (1989) Oestrogen replacement therapy and cardiovascular disease in post-menopausal women. A review. Maturitas 11: 259–274. 2693914 10.1016/0378-5122(89)90023-6

[2] Alessi MC, Juhan-Vague I (2008) Metabolic syndrome, haemostasis and thrombosis. Thromb Haemost 99: 995–1000. 18521499 10.1160/TH07-11-0682

[3] Lakka HM, Laaksonen DE, Lakka TA, Niskanen LK, Kumpusalo E, et al (2002) The metabolic syndrome and total and cardiovascular disease mortality in middle-aged men. JAMA 288: 2709–2716. 12460094 10.1001/jama.288.21.2709

[4] Freeman MS, Mansfield MW, Barrett JH, Grant PJ (2003) Insulin resistance: an atherothrombotic syndrome. The Leeds family study. Thromb Haemost 89: 161–168. 12540966

[5] Lim S, Park KS, Lee HK, Cho SI, Korean National H, et al (2005) Changes in the characteristics of metabolic syndrome in Korea over the period 1998–2001 as determined by Korean National Health and Nutrition Examination Surveys. Diabetes Care 28: 1810–1812. 15983345 10.2337/diacare.28.7.1810

[6] Chowdhury TA, Boucher BJ, Hitman GA (2009) Vitamin D and type 2 diabetes: Is there a link? Prim Care Diabetes 3: 115–116. 19395331 10.1016/j.pcd.2009.03.004

[7] Awumey EM, Mitra DA, Hollis BW, Kumar R, Bell NH (1998) Vitamin D metabolism is altered in Asian Indians in the southern United States: a clinical research center study. J Clin Endocrinol Metab 83: 169–173. 9435436 10.1210/jcem.83.1.4514

[8] International Physical Activity Questionnaire (IPAQ) Core Group (2005) Guidelines for data processing and analysis of the International Physical Activity Questionnaire–Short and Long Forms. Available: http://www.ipaq.ki.se/scoring.pdf. Accessed 25 October, 2013.

[9] Soules MR, Sherman S, Parrott E, Rebar R, Santoro N, et al (2001) Executive summary: Stages of Reproductive Aging Workshop (STRAW). Fertil Steril 76: 874–878. 11704104 10.1016/s0015-0282(01)02909-0

[10] Holick MF (2007) Vitamin D deficiency. N Engl J Med 357: 266–281. 17634462 10.1056/NEJMra070553

[11] Dawson-Hughes B, Heaney RP, Holick MF, Lips P, Meunier PJ, et al (2005) Estimates of optimal vitamin D status. Osteoporos Int 16: 713–716. 15776217 10.1007/s00198-005-1867-7

[12] Choi SH, Kim DJ, Lee KE, Kim YM, Song YD, et al (2004) Cut-off value of waist circumference for metabolic syndrome patients in Korean adult population. J Korean Soc Study Obes 13: 53–60.

[13] Lu L, Yu Z, Pan A, Hu FB, Franco OH, et al (2009) Plasma 25-hydroxyvitamin D concentration and metabolic syndrome among middle-aged and elderly Chinese individuals. Diabetes Care 32: 1278–1283. 19366976 10.2337/dc09-0209 PMC2699709

[14] Ford ES, Ajani UA, McGuire LC, Liu S (2005) Concentrations of serum vitamin D and the metabolic syndrome among U.S. adults. Diabetes Care 28: 1228–1230. 15855599 10.2337/diacare.28.5.1228

[15] Hypponen E, Boucher BJ, Berry DJ, Power C (2008) 25-hydroxyvitamin D, IGF-1, and metabolic syndrome at 45 years of age: a cross-sectional study in the 1958 British Birth Cohort. Diabetes 57: 298–305. 18003755 10.2337/db07-1122

[16] Cheng S, Massaro JM, Fox CS, Larson MG, Keyes MJ, et al (2010) Adiposity, cardiometabolic risk, and vitamin D status: the Framingham Heart Study. Diabetes 59: 242–248. 19833894 10.2337/db09-1011 PMC2797928

[17] Kim S, Limb J, Kyec S, Joung H (2012) Association between vitamin D status and metabolic syndrome risk among Korean population: based on the Korean National Health and Nutrition Examination Survey IV-2, 2008. Diabetes Res Clin Pract 96: 230–236. 22309788 10.1016/j.diabres.2012.01.001

[18] George JA, Norris SA, van Deventer HE, Crowther NJ (2013) The association of 25 hydroxyvitamin D and parathyroid hormone with metabolic syndrome in two ethnic groups in South Africa. PLoS One 8: e6128. 10.1371/journal.pone.0061282 PMC3626636 23596520

[19] Song J, Kim E, Shin C, Kim SS, Lee HK, et al (2004) Prevalence of the metabolic syndrome among South Korean adults: the Ansan Study. Diabet Med 21: 1064–1065. 15384966 10.1111/j.1464-5491.2004.01399.x

[20] Stoney CM, Owens JF, Guzick DS, Matthews KA (1997) A natural experiment on the effects of ovarian hormones on cardiovascular risk factors and stress reactivity: bilateral salpingo oophorectomy versus hysterectomy only. Health Psychol 16: 349–358. 9237087 10.1037//0278-6133.16.4.349

[21] Park YW, Zhu S, Palaniappan L, Heshka S, Carnethon MR, et al (2003) The metabolic syndrome: prevalence and associated risk factor findings in the US population from the Third National Health and Nutrition Examination Survey, 1988–1994. Arch Intern Med 163: 427–436. 12588201 10.1001/archinte.163.4.427 PMC3146257

[22] Park HS, Zhu S, Palaniapan L, Heshka S, Carnethono MR, et al (2003) The metabolic syndrome: prevalence and associated risk factor findings in the US population from the Third National Health and Nutrition Examination Survey, 1998–1994. Arch Intern med 163: 427–436. 12588201 10.1001/archinte.163.4.427 PMC3146257

[23] Oh JY, Hong YS, Sung YA, Barrett-Connor E (2004) Prevalence and factor analysis of metabolic syndrome in an urban Korean population. Diabetes Care 27: 2027–2032. 15277435 10.2337/diacare.27.8.2027

[24] Dayspring TD (2011) Understanding hypertriglyceridemia in women: clinical impact and management with prescription omega-3-acid ethyl esters. Int J Womens Health 3: 87–97. 21445284 10.2147/IJWH.S16702 PMC3061852

[25] Mascarenhas-Melo F, Sereno J, Teixeira-Lemos E, Ribeiro S, Rocha-Pereira P, et al (2013) Markers of increased cardiovascular risk in postmenopausal women: focus on oxidized-LDL and HDL subpopulations. Dis Markers 35: 85–96. 24167352 10.1155/2013/724706 PMC3774979

[26] Schnatz PF, Nudy M, O'Sullivan DM, Ethun K, Appt SE, et al (2011) Identification of a mechanism for increased cardiovascular risk among individuals with low vitamin D concentrations. Menopause 18: 994–1000. 21593696 10.1097/gme.0b013e318212539d PMC3683239

[27] Rye KA, Bursill CA, Lambert G, Tabet F, Barter PJ (2009) The metabolism and anti-atherogenic properties of HDL. J Lipid Res 50: 195–200. 10.1194/jlr.R800034-JLR200 PMC2674714 19033213

[28] Matsuura F, Wang N, Chen W, Jiang XC, Tall AR (2006) HDL from CETP-deficient subjects shows enhanced ability to promote cholesterol efflux from macrophages in an apoE- and ABCG1-dependent pathway J Clin Invest. 116: 1435–1442. 10.1172/JCI27602 PMC1451209 16670775

[29] Snijder MB, Lips P, Seidell JC, Visser M, Deeg DJ, et al (2007) Vitamin D status and parathyroid hormone levels in relation to blood pressure: a population-based study in older men and women. J Intern Med 261: 558–565. 17547711 10.1111/j.1365-2796.2007.01778.x

[30] Hintzpeter B, Mensink GBM, Thierfelder W, Muller MC, Scheidt-Nave C (2008) Vitamin D status and health correlates among German adults. Eur J Clin Nutr 62: 1079–1089. 17538533 10.1038/sj.ejcn.1602825

[31] Wang TJ, Pencina MJ, Booth SL, Jacques PF, Ingelsson E, et al (2008) Vitamin D deficiency and risk of cardiovascular disease. Circulation 117: 503–511. 18180395 10.1161/CIRCULATIONAHA.107.706127 PMC2726624

[32] Pasco JA, Henry MJ, Nicholson GC, Brennan SL, Kotowicz MA (2009) Behavioural and physical characteristics associated with vitamin D status in women. Bone 44: 1085–1091. 19264157 10.1016/j.bone.2009.02.020

[33] Jorde R, Figenschau Y, Emaus N, Hutchinson M, Grimnes G (2010) Serum 25-hydroxyvitamin D levels are strongly related to systolic blood pressure but do not predict future hypertension. Hypertension 55: 792–798. 20065152 10.1161/HYPERTENSIONAHA.109.143990

[34] Seo JA, Eun CR, Cho H, Lee SK, Yoo HJ, et al (2013) Low vitamin D status is associated with nonalcoholic Fatty liver disease independent of visceral obesity in Korean adults. PLoS One 8(10): e75197. 24130687 10.1371/journal.pone.0075197 PMC3793981

[35] Holick MF (2004) Vitamin D: importance in the prevention of cancers, type 1 diabetes, heart disease, and osteoporosis. Am J Clin Nutr 79: 362–371. 14985208 10.1093/ajcn/79.3.362

